# Evaluation of the Use of Elicitors for the Production of Antioxidant Compounds in Liquid Cultures of *Ganoderma curtisii* from Costa Rica

**DOI:** 10.3390/molecules27134265

**Published:** 2022-07-02

**Authors:** Catalina Rosales-López, Felipe Arce-Torres, Mariana Monge-Artavia, Miguel Rojas-Chaves

**Affiliations:** Centro de Investigación en Biotecnología, Instituto Tecnológico de Costa Rica (ITCR), Cartago 30101, Costa Rica; felipe14arce@gmail.com (F.A.-T.); marige97@gmail.com (M.M.-A.); mirojas@itcr.ac.cr (M.R.-C.)

**Keywords:** pH, peptone, ORAC, growth regulators, ethanol, salts

## Abstract

The use of substances or conditions as elicitors can significantly increase the production of secondary metabolites. In this research, the effects of different elicitors on the production of antioxidant secondary metabolites were evaluated in a strain of *Ganoderma* sp. The elicitors tested were pH changes in different growth phases of the fungus (pH 3, 5.5 and 8), different concentrations of peptone as a nitrogen source (1 g/L and 10 g/L), and the addition of chemical agents to the culture medium (ethanol, growth regulators, and salts). The alkaline pH during the stationary phase and the high availability of nitrogen were effective elicitors, producing cultures with higher antioxidant activity (37.87 g/L and 43.13 g/L dry biomass) although there were no significant differences with other treatments.

## 1. Introduction

The genus *Ganoderma* has been used for many years in diverse cultures for its wide range of nutraceutical and medicinal benefits [1]. *Ganoderma* belongs to the polypore basidiomycetes, the most studied species are *Ganoderma lucidum*, *Ganoderma adspersum*, and *Ganoderma applanatum*. *Ganoderma lucidum* is used in infusions in Asian countries such as Japan and China [2].

Benefits and bioactive effects reported for this genus include anti-inflammatory, antioxidant [3], heptaprotective, immunomoduladatory, antidiabetic, neuroprotector, and antibacterial activity. These properties are attributed to the more than 430 secondary metabolites present in the biomass, particularly triterpenes and polysaccharides [4,5].

In vitro culture of *Ganoderma* sp. is extremely useful; development of the fungus in a controlled environment allows the study of physiological and anatomical properties [6] and facilitates subsequent production of bioactive compounds through exposure to stress (irradiation, water stress, parasites, and increase or decrease in concentrations of culture media, among others) [7].

Elicitors are substances or conditions that promote the production of bioactive metabolites as defense mechanisms. The use of elicitors to stimulate production of secondary metabolites can magnify the bioactive effects of *Ganoderma* sp. [8]. Elicitors can be classified as biotic (fungal or bacterial compounds, mycotoxins, living organisms) or abiotic, which can be chemical (mineral salts, heavy metals), physical (UV radiation, osmotic stress, hydrodynamic and thermal stress) or hormonal. The type of elicitor used will determine the response generated in the production of specific metabolites [9].

The objective of the present study was to evaluate the effect of elicitors on the production of secondary metabolites with antioxidant activity, in a strain of *Ganoderma curtisii* collected in Costa Rica.

## 2. Methods

### 2.1. Fungal Culture

The fungus, identified molecularly and morphologically as *Ganoderma curtisii*, its strain was provided by the Fungal Culture Collection of the Escuela de Ingeniería Forestal at the Instituto Tecnológico de Costa Rica (Accession number CIIBI-007A, Biodiversity permit R-CM-ITCR-005-2019-OT).

#### 2.1.1. Culture Maintenance

The fungus was maintained in solid potato-dextrose agar (PDA) in petri plates at 37 °C and subcultured weekly.

#### 2.1.2. Inoculum Preparation

Preinoculum *Ganoderma curtisii* cultures were prepared by inoculating liquid media with three agar disks from 8-day cultures. The liquid medium consisted of glucose (30 g/L), yeast extract (5 g/L), peptone (5 g/L), K_2_HPO_4_ (0.5 g/L), KH_2_PO_4_ (0.5 g/L), MgSO_4_·7H_2_O (0.5 g/L), vitamin B1 (0.05 g/L), and olive oil^®^ (1% *v*/*v*). The pH was pH 5.5 (Rosales-López et al. sf) [10]. Cultures were incubated at 27 °C with agitation (100 rpm) for one week.

### 2.2. Elicitor Experiments

Suspensions of Ganoderma curtisii were prepared in 50 mL of medium in 250 mL flasks by inoculating to 1% (*m*/*v*) using preinoculum [10]. The pH of the culture media was 5.5 prior to autoclaving. Cultures were maintained at 27 °C with agitation on a shaker at 100 rpm for 14 or 21 days, depending on each treatment. Elicitor treatments are described in Table 1. Each treatment was applied in triplicate. Means are reported for each treatment. 

#### 2.2.1. Effect of pH

The effect of pH was evaluated at each of the fungal growth phases: exponential and stationary. Initial cultures of *Ganoderma curtisii* were prepared and grown at pH 5.5 for one week (exponential phase, treatments A and B) or two weeks (stationary phase, treatments C and D). On day 8 for A and B and on day 15 for C and D, the mycelium was filtered and transferred to fresh culture media adjusted to pH 8 (treatments A and C) or pH 3 (treatments B and D). Other initial cultures were allowed to grow at pH 5.5 for two weeks (treatment E) or three weeks (treatment F) without modification of the pH (Table 1). 

#### 2.2.2. Effect of Growth Regulators 

To evaluate the effect of growth regulators, 1 mg/L of growth regulator was added to one-week cultures of *Ganoderma curtisii*. Treatments were BAP (benzylaminopurine, treatment G), AIA (indole acetic acid, treatment H), and AG3 (gibberellic acid, treatment I). All treatments were continued for two weeks after adding the growth regulator (Table 1).

#### 2.2.3. Effect of Nitrogen Availability

The effect of nitrogen availability was evaluated using two levels of peptone added to the culture medium. Initial cultures were grown for 8 days and then transferred to fresh media with limited nitrogen (1 g/L, treatment J), or to nitrogen-rich media (10 g/L, treatment K). Cultures were maintained for two additional weeks (Table 1). 

#### 2.2.4. Effect of Ethanol and Salts in the Medium

To evaluate the effects of ethanol and salts on fungal growth, ethanol (95% *v*/*v*), NaCl, MnCl_2_, and CaCl_2_ were added to fungal cultures. Ethanol 1.5% (*v*/*v*) was added to one-week cultures (treatment L) and they were maintained for two additional weeks. Salts were added to two-day cultures (100 mM NaCl, treatment M; 10 mM MnCl_2_, treatment N; 10 mM CaCl_2_, treatment O), and they were maintained for 19 additional days (Table 1).

### 2.3. Analysis of Results

#### 2.3.1. Estimation of Biomass

After each experiment, the contents of each flask were vacuum filtered to separate the medium from the mycelium. The mycelium was lyophilized and ground for extraction.

Fresh biomass concentration (g/L), dry biomass concentration (g/L), and yield (g/g) were determined for each treatment (Table 1).

Cultures without elicitor treatments were grown for 2 or 3 weeks as controls. 

#### 2.3.2. Estimation of Antioxidant Activity using the ORAC Method

##### Extraction of Bioactive Compounds

Reagent grade methanol (3 mL) was added to 75 mg of each sample. Samples were placed in an ultrasonic bath (Fisher-Scientific, Chicago, IL, USA) for 10 min, then centrifuged for 5 min at 2000 rpm (Eppendorf 5810R, Hamburg, Germany). The supernatant was collected, and the extraction was repeated twice. The supernatant collected from the three extractions was brought to 10 mL with ultra-pure water (Milli-Q^®^).

Antioxidant activity was determined using the method described by Huang, D. et al. (2002) [11] with modifications for *Ganoderma curtisii*. The extracts were diluted 1:16 with phosphate buffer and the final reaction volume was 200 μL. Dissolution buffer was used instead of antioxidant as a blank. Five Trolox solutions were prepared (20%, 40%, 50%, 80%, and 100%). The area under the fluorescence decay curve was calculated for the blank and the sample, and the difference was expressed as micromole Trolox equivalents per 100 mL (μmol ET/100 mL). As a control for comparison with treatments, the antioxidant activity of the fruiting body collected in the natural environment was quantified.

### 2.4. Statistical Analysis

Analysis of variance (ANOVA) was used to determine significant differences and Tukey’s test was used to group data with a confidence level of 95%. All analyses were performed using the statistical software Minitab.

## 3. Results and Discussion

This research was carried out with a strain of *Ganoderma* collected in the forests of Costa Rica, a tropical country with a great biodiversity, but the bibliographic review was taken from studies carried out with a different strain collected in China, Japan, and India. Therefore, differences in the results were expected, since geographical differences affect the strain behavior, its chemical composition, and its management and adaptation to a certain culture medium. In addition, the growth phase was prioritized in order to elicit during this research.

### 3.1. Effect of Elicitors on Biomass Production

Biomass and bioactive compounds produced in untreated fungal cultures (without elicitors) and bioactive compounds in field-collected fruiting bodies are shown in Table 2.

The fresh biomass concentration in cultures grown for three weeks in liquid media was higher than in the two-week cultures. This difference was due to greater hydration with longer exposure; dry biomass concentration in two- and three-week treatments was not significantly different. Prior studies of growth kinetics showed that after 14 days, the fungus enters the stationary phase (Rosales-López et al. sf) [10] in which more secondary metabolites are produced [19]. In untreated cultures, antioxidant activity increased from 97.52 in the exponential phase to 169.08 μmol ET/g dry weight in the stationary phase due to the presence of bioactive compounds, possibly ganoderic acids [20] or polysaccharides [9].

#### 3.1.1. Effect of pH

The effect of exposure to acidic (pH 3) or basic (pH 8) conditions on biomass production and antioxidant activity is shown in Table 1 and Table 2. Biomass dry weight was greater in two- and three-week cultures at pH 3 and pH 8 than at pH 5.5 (E and F), while Jayasinghe, C. et al. (2008) [21] reported the most suitable pH for mycelial growth was found at pH 5 for *Ganoderma*.

pH may affect cell membrane function, cell morphology, and structure. The solubility of salts, the ionic state of substrates, the uptake of various nutrients, and product biosynthesis may be affected, too. In general, cells grow in optimal conditions within a certain pH range, but the metabolite formation is also often affected by different pH levels [12].

#### 3.1.2. Effect of Growth Regulators

The effect of plant growth regulators on fungal growth did not show significant difference. Relative to the three-week control cultures, biomass was similar in cultures treated with growth regulators (Table 2).

Biomass production was higher in treatments with AG3 (27,732 g/L) than with AIA (25,412 g/L) or BAP (26,372 g/L). These results differ from those obtained by Xu, Y. et al. (2013) [16], who reported greater growth using BAP (35 g/L approximately), followed by AIA (30 g/L approximately), and lower growth with AG3 (20 g/L approximately). Some differences between the two experiments were the culture techniques for *Ganoderma*, the growth period (10 days), and the application time of the regulators, which in their case, were applied to the initial cultures (adaptation phase). In this research, the application of the regulators occurred during the exponential phase of fungal growth, allowing growth to be prolonged, and they were left at the end of the stationary phase, enhancing the production of bioactive compounds.

Growth regulators in fungi are used to improve growth conditions, based on the use of phytohormones, controlling the production of secondary metabolites, growth time, decrease of the concentration of pathogens, etc., which are naturally difficult processes to regulate in a conventional culture medium [13].

#### 3.1.3. Effect of Nitrogen Availability

Addition of peptone to the culture medium as a nitrogen source at a low or high concentration (Table 2) increased biomass production relative to the three-week control culture (Table 2). Although there were no significant differences in the results, the increase in nitrogen concentration in the exponential phase allowed a higher biomass production.

The carbon-to-nitrogen ratio (C/N) is an important factor affecting mycelium and fruiting body formation in medicinal mushrooms [6]. For *Ganoderma lucidum*, the effect of various nitrogen sources on its growth has been studied, finding that peptone and yeast extract promote higher growth and metabolite production compared to other inorganic nitrogen sources [6].

On the other hand, researchers [12] surmised that peptone may have some growth inhibitory components for *Ganoderma lucidum*. However, as the peptone concentration was reduced in lactose-containing cultures, the biomass production was increasing, showing an inverse effect of growth and peptone concentration which does not suggest that peptone has inhibitory effects, but yeast extract is a better nitrogen source.

#### 3.1.4. Effect of Ethanol and Salts in the Culture Medium

Comparison of the dry biomass shown in Table 2 with the three-week control cultures showed minimal reduction in the reduced growth of *Ganoderma curtisii* on media with added ethanol (L treatment), NaCl (M treatment), MnCl_2_ (N treatment), or CaCl_2_ (O treatment). That is, there was no significant difference in biomass growth upon addition of these elicitors.

The limited growth may indicate that these compounds generated negative stress in the fungus.

### 3.2. Effect of Elicitors on Production of Compounds with Antioxidant Activity

Table 2 shows the antioxidant capacity of *Ganoderma curtisii* exposed to elicitor treatments and their respective controls. Significantly different treatments, according to Tukey’s pairwise comparison, are indicated by different letters.

#### 3.2.1. Effect of pH

In the present study, antioxidant activity was higher when the pH was varied, but not significantly higher than in the other treatments (Table 2). The elicitation of the fungus by varying the pH of the medium was effective to appreciate an exaggerated increase in antioxidant activity at pH 8 (267.6 μmol TE*/g dry sample), compared to pH 5.5, pH 3, and the control (169 μmol TE*/g dry sample). The fungus was stressed due to an abrupt pH variation in its medium.

Antioxidant activity was higher in the three-week cultures, confirming that production of secondary metabolites was higher during the stationary phase of fungal growth. On the other hand, Islas-Santillán, M. et al. (2017) [3] found that pH affects the production of bioactive compounds in different ways in *Ganoderma* sp., the production of intracellular polysaccharides is favored at pH 5.5–7, while extracellular polysaccharides are favored by a low pH, and ganoderic acids are favored at pH 5.5–6.5 [3].

Accumulation of these metabolites often occurs under stress, elicitors or signaling molecules can be used to increase their production as well. Environmental factors or culture conditions (in vitro) such as temperature, humidity, light intensity, water supply, pH, minerals, and CO_2_ influence fungal growth and the production of secondary metabolites. Additionally, environmental conditions can cause adverse effects on growth and productivity [9]. Moreover, the modification of pH at different growth stages helps to stress the microorganism to naturally produce the bioactive compounds of interest.

#### 3.2.2. Effect of Growth Regulators

There was no significant difference between the three growth regulators tested (BAP, AIA, and AG3), the biomass growth was very similar, but the production of antioxidant secondary metabolites was slightly more enhanced in all growth regulator treatments relative to the three-week control, although not significantly so. Treatment G with an increase in antioxidant activity of 22.8 μmol TE*/g dry sample relative to the control, treatment H had a difference of 39.92 μmol TE*/g dry sample, and treatment I had a 31.92 μmol TE*/g dry sample difference to the control. Therefore, these elicitors were successful. Antioxidant production was higher in the *Ganoderma curtisii* cultures treated with the regulator AIA. The function of AIA is to induce growth by rapidly stimulating the synthesis of cell wall components of growing cells [13] during the stationary phase by assisting in the production of bioactive compounds.

#### 3.2.3. Effect of Nitrogen Availability

The microorganism was stressed by increasing the peptone concentration in the medium as an increase in the antioxidant capacity of the culture extracts is perceived, but not significantly, since it increased 88 μmol TE*/g dry sample antioxidant activity with respect to the treatment with low nitrogen concentration. It can be said that both conditions stress the microorganism, producing secondary metabolites. In the study of Thao, C., and Tien, L. (2017) [13], limiting nitrogen resulted in greater metabolite production.

#### 3.2.4. Effect of Ethanol and Salts

The antioxidant capacity of extracts from cultures treated with 95% ethanol (treatment L) and the three-week control cultures showed no significant differences (Table 2), although the activity was lower in the L treatment. This result differed from that obtained by Yang, H., Wu, T., and Zhang, K. (2004) [15] in which the addition of ethanol increased biomass and metabolite production. In the present study, the negative effect of ethanol on the culture may have been due to the inhibition of metabolite production, or the concentration of the elicitor may have been too low to generate the expected result.

With respect to the added salts, the antioxidant activity detected in treatments with NaCl were not statistically different than the standard (Trolox) (Table 2). This result was not expected, Xu, Y., Xia, X. and Zhong, J. (2013) [18] found a positive effect of this salt on the production of ganoderic acids which have antioxidant activity. From the salts tested, MnCl_2_ generated higher antioxidant activity than the other salts, the three-week control, and the fruiting body. On the other hand, CaCl_2_ produced less antioxidant activity than the three-week control. This differed from results obtained by Xu, Y. and Zhong, J. (2012) [18], who found that this salt favors the production of antioxidant secondary metabolites.

There is a lack of information about the mechanism of how it acts, but Xu, Y. and Zhong, J. (2012) [18] acknowledged that Ca^2+^ signals were found to be important in elicitor-induced secondary metabolite accumulation.

## 4. Conclusions

Elicitors affected the production of biomass and secondary metabolites. When the fungus was elicited by varying the culture conditions from pH 5.5 to alkaline (pH 8) and increasing the nitrogen concentration, increased biomass growth and antioxidant activity were observed. Extracts from cultures produced under these conditions showed the highest antioxidant capacity, which indicates greater production in vitro of compounds of interest.

Comparison of all elicitor treatments showed that antioxidant capacity, and therefore production of metabolites of interest, was highest in three-week cultures exposed to pH 8 (treatment C) and in cultures with high available nitrogen (treatment K). These elicitors are easy to obtain, economical, and simple to handle.

## Figures and Tables

**Table 1:** List of elicitor treatments and comparison of yields with similar references.

Treatment	Elicitor	Duration of Experiment (Weeks)	Yield of Dry Biomass (±0.0001 g/L)	Reference	Estimated Yield of Dry Biomass (g/L)
A	Alkaline pH (8) exponential phase	Two	28.3460	[12]	16.00 ± 0.37 *
B	acid pH (3) exponential phase	Two	26.9240	[12]	13.8 ± 0.01 *
C	Alkaline pH (8) stationary phase	Three	37.8740	[12]	16.00 ± 0.37 *
D	acid pH (3) stationary phase	Three	34.4360	[12]	13.8 ± 0.01 *
E	pH 5.5 exponential phase	Two	19.6340	[12]	15.5 ± 0.3 *
F	pH 5.5 stationary phase	Three	19.0840	[12]	15.5 ± 0.3 *
G	Growth regulator BAP	Three	26.3720	[13]	35.0 ± 0.1 **
H	Growth regulator AIA	Three	25.4120	[13]	30.0 ± 0.1 **
I	Growth regulator AG_3_	Three	27.7320	[13]	20.0 ± 0.1 **
J	Low source of nitrogen	Three	42.3060	[14]	8.88 ± 0.01 *
K	High source of nitrogen	Three	43.1260	[14]	10.83 ± 0.01 *
L	95% ethanol	Three	22.4600	[15]	9.56 ± 0.01 ***
M	Sodium chloride	Three	24.6520	[16]	17.39 ± 0.47 ***
N	Manganese chloride	Three	22.9520	[17]	-
O	Calcium chloride	Three	24.2400	[18]	16.79 ± 0.89 ***

* Biomass per week, ** Biomass after 10 days growth *** Biomass after two weeks of growth.

**Table 2:** Fresh biomass, dry biomass, and antioxidant activity of untreated in vitro *Ganoderma* sp. Cultures (C2W, C3W, FB), treated with high and low pH for 2 weeks and three weeks (A–F), growth regulators (G–I), high and low nitrogen availability (J,K) ethanol (L), salts in the medium (M–O) and the *Ganoderma* sp. fruiting body.

Treatment	Fresh Biomass (g/L)	Dry Biomass (g/L)	Antioxidant Activity (μmol TE*/g Dry Sample)	Duration of Culture
Two weeks control (C2W)	218.6 ^e,f^	22.62 ^a (**)^	97.52 ^c,d,e,f,g,h^	2 weeks
Three weeks control (C3W)	597.6 ^a^	28.76 ^a^	169.08 ^a,b,c,d,e,f,g^	3 weeks
Fruiting body (FB)	-	-	124.81 ^b,c,d,e,f,g,h^	-
A	215.906 ^e,f^	28.346 ^b,c,d^	95.84 ^d,f,g,h^	2 weeks
B	176.892 ^f^	26.924 ^c,d^	73.95 ^g,h^	2 weeks
C	411.664 ^a,b,c^	37.874 ^a,b,c^	267.60 ^a^	3 weeks
D	347.732 ^b,c,d^	34.436 ^a,b,c^	187.14 ^a,b,c^	3 weeks
E	297.776 ^d,e^	19.634 ^d^	185.06 ^a,b^	2 weeks
F	521.812 ^a^	19.084 ^d^	28.96 ^h^	3 weeks
G	508.092 ^a^	26.372 ^b,c,d^	191.88 ^a,b,c,d,e,f^	3 weeks
H	545.666 ^a^	25.412 ^c,d^	209.70 ^a,b,c^	3 weeks
I	526.806 ^a^	27.732 ^a,b,c,d^	201.67 ^a,b,c,d^	3 weeks
J	483.826 ^a,b^	42.306 ^a,b^	174.15 ^a,b,c,d,e,f,g^	3 weeks
K	484.360 ^a^	43.126 ^a^	262.15 ^a^	3 weeks
L	412.412 ^a,b,c,d^	22.46 ^c,d^	136.13 ^b,c,d,e,f,g,h^	3 weeks
M	324.506 ^c,d,e^	24.65 ^c,d^	9.94 ^e,f,g,h^	3 weeks
N	500.046 ^a^	22.95 ^c,d^	192.86 ^a,b,c,d^	3 weeks
O	390.926 ^b,c^	24.24 ^c,d^	106.96 ^b,c,d,e,f,g,h^	3 weeks

* TE: Trolox equivalent. ** Different letters in the same column indicate statistically significant differences.

## Data Availability

Data is contained within the article.

## Sample Availability

Samples of the compounds are not available from the authors.
